# Retraction: EPO Receptor Gain-of-Function Causes Hereditary Polycythemia, Alters CD34^+^ Cell Differentiation and Increases Circulating Endothelial Precursors

**DOI:** 10.1371/journal.pone.0230279

**Published:** 2020-03-04

**Authors:** The PLOS ONE Editors

Following the publication of this article [1], concerns have been raised regarding panels presented in Figs 3, 4, 5 and 6:

- In Fig 3C there appear to be horizontal and vertical edges around the bands presented in lanes 3 and 4. In addition, the bands in lane 3 and lane 5 appear similar.
- In Fig 3C, the top bands in lane 4 and lane 6 appear similar. In addition, the bottom band in lane 4 appears similar to the top band in lane 4, when rotated 180°.
- In Fig 4B, the bands in lane 1 and 2 representing WT-EPOR in the Con and MutEPOR samples (7 days) appear similar.
- In Fig 4D, the bands in lane 2 and 3 representing MutEPOR in the MutEPOR LnLL + and–cells appear similar.
- Lanes 3 and 4 in Fig 4B representing 10 day WT-EPOR and MutEPOR look similar to lanes 1 and 2 in Fig 5B representing EPOR and MutEPOR.
- Lanes 1 and 2 in Fig 5D representing STAT5 look similar to lanes 2 and 3 in Fig 4B representing GlyA when rotated 180°.
- There appear to be horizontal edges surrounding the pErk1 and pErk2 bands in Fig 6A, and the background granularity surrounding the bands appears dissimilar to the background of the rest of the panel.
- There appear to be horizontal and vertical edges surrounding the Erk1 and Erk2 bands in Fig 6A, and the background granularity surrounding the bands appears dissimilar to the background of the rest of the panel
- There is a rectangular grey area located between the control actin band and the Mutated EpoR actin band in Fig 6D which does not match the overall background of the panel.

The underlying data for the western blots and PCR results of concern are not available. The authors provided replicate data and data from new experiments in support of Figs 3, 4, 5, and 6, but these did not resolve the concerns about the published figures.

In light of the concerns affecting multiple figure panels that question the integrity of these data, the *PLOS ONE* Editors retract this article.

SP, LR, FR, AB, MDC, ARM, IMP, FB, DY, and FDR agree with retraction. DR did not agree with the retraction. JTP did not confirm agreement or disagreement with the retraction decision. VC, MF, AT, ACS, BN, AO, GA, and PM did not respond directly or could not be reached. SP, LR, FR, AB, MDC, ARM, IMP, FB, DR and FDR stand by the conclusions reported in the paper.
